# Supplementary material for: Determinants of chronic energy deficiency among non-pregnant and non-lactating women of reproductive age in rural Kebeles of Dera District, North West Ethiopia, 2019: Unmatched case control study
Source: PLoS One. 2020 Oct 29;15(10):e0241341. doi: 10.1371/journal.pone.0241341 (PMC7595398; doi:10.1371/journal.pone.0241341)
Supplement: S1 File — (DOCX) [file pone.0241341.s001.docx]

Wollo University

College of Medicine and Health Science

School of Public Health

Questionnaires to assess the determinants of chronic energy deficiency among non-pregnant and non-lactating women in Dera District, South Gondar zone, 2019፡ English version

| Sr. No | Questions | Response | code | |
| --- | --- | --- | --- | --- |
| 1. Socio- socio economic and demographic characteristics | | | | |
| 1.1 | Age | ………………… years |  | |
| 1.2 | Marital status | 1. Married  2. Divorced  3. Widowed  4. single |  | |
| 1.3 | Your age at first marriage | ……….Years |  | |
| 1.4 | Religion | 1. Orthodox  2. Muslim  3. Protestant  4. Catholic  5. others, specify--------------- |  | |
| 1.5 | Ethnicity | 1. Amhara  2. Oromo  3. Tigre  4. Others……. |  | |
| 1.6 | Educational status of women | 1. Illiterate  2. can read and write  3. primary education and above |  | |
| 1.7 | Head of the house hold? | 1. Farmer  2. Women  3. Other, specify,___________ |  | |
| 1.8 | Occupational statuses of house hold head? | 1. Father  2. Merchant  3. Other………. |  | |
| 1.9 | Educational status of the head of the households? | 1. Illiterate  2. can read and write  3. primary education and above |  | |
| 1.10 | Your annual income | ----------- quntals(100kg) |  | |
| **2.Availability of home gardening** | | | | |
| 2.1 | Do you have home gardening? | 1. Yes  2. No , | If no skip toQn.4.1 | |
| 2.2 | What do you cultivate in it? | 1. Vegetable  2. Fruit  3. Both  4. Other, specify_____ |  | |
| 2.3 | For what purpose do you grow the vegetables and/or fruit? | 1. For selling  2. For home consumption only  3. For both purpose  4. Other, specify_________ |  | |
| 1. **Individual diet diversity and meal frequncy**: - In the past 24 hours, if the participant consumed at least one of the food group from the list, write “1” in the box and if not write “0”. | | | | |
|  | Food group | Examples | code | |
| 3.1 | Cereals | corn/maize, wheat, sorghum or any other grains or foods made from these (e.g. bread, enjera, | Yes= 1  No= 0 | |
| 3.2 | Vitamin A rich fruits and vegetables | mango. carrots, or sweet potatoes that are orange inside |  | |
| 3.3 | Tubers and roots | potatoes, beetroot or other foods made from roots |  | |
| 3.4 | Dark green leafy vegetables | dark green/leafy vegetables, cabbage, etc. |  | |
| 3.5 | Otherfruits and vegetables | other vegetables (e.g. tomato, onion) |  | |
| 3.6 | Fish meats meats | lamb, goat, chicken, fish |  | |
| 3.7 | Eggs | chicken, egg |  | |
| 3.8 | Legumes, nuts | beans, peas, lentils, nuts |  | |
| 3.9 | Milk and milk products | milk, cheese, yogurt or other milk products |  | |
| 3.10 | In the past 24 hours how many times did you serve meals in a day? | 1. Less than three meals  2. 3 meals  3. 4 meals  4. > 4 meals |  | |
| 1. **. Access to safe drinking water and latrine facility** | | | | |
| 4.1 | Where did you get water for drinking? | - Public tap - Spring - Surface water - Other, specify _______ |  | |
| 4.2 | Do you treat water for drinking? | 1. Yes  2. No ,If no skip to Qn. 5.4 |  | |
| 4.3 | How do you treat water? | 1. Boiling  2. Filter through a cloth  3. Other specify ______ |  | |
| 4.4 | How long does it take to go there, to get water, and come back? | _________ Minute |  | |
| 4.5 | How many times travel to fetch water every day? | 1. Once a day  2. Twice a day  3. Three and more times |  | |
| 4.6 | Do you have a toilet facility in your home? | 1.yes  2. no |  | |
| 4.7 | Do you wash your hand after latrine? | 1. Yes  2. No |  | |
| 4.8 | If you don’t have a toilet in your home, where do you use? | 1. Public toilet  2. Open field  3. Other, specify_________ |  | |
| 1. **. Maternal care and health related factors** | | | | |
| 5.1 | Number of gravidity? | ----------- |  | |
| 5.2 | Number of parity? | ------------- |  | |
| 5.3 | Age of last child? | ------------month |  | |
| 5.4 | Did you take ANC follow up during your last pregnancy | 1. Yes 2. No |  | |
| 5.5 | Number of family members in the household? | ___________ |  | |
| 5.6 | Did you use modern family planning techniques for birth spacing | 1. Yes 2. No |  | |
| 5.7 | Do you have a history febrile illness in the past one month? | 1. Yes  2. No , If no, skip to Qn. 6.1 |  | |
| 5.8 | For how many days did the illness stay? | 1. Less than a weeks  2. 1-2 weeks  3. 3-4 weeks  4. Other _____ weeks |  | |
| 5.9 | When do you do recovered from the illness? | 1. Within this week  2. Before two week  3. Before three week  4. Other specify_______ |  | |
| 1. **Anthropometric data** | | | | |
| 6.1 | Weight in kilogram | measurment1……kg, measurment2…….kg | |  |
| 6.2 | Height in meter | measurement1…..m measurment2……m | |  |

በደራ ወረዳ ገጠር ቀበሌዎች በመዉለድ እድሜ ክልል ያሉ ነፍሰ-ጡር ያልሆኑና የማያጠቡ እናቶች ለምግብ እጥረት ስለሚያጋልጡ ምክንያቶችና ተያያዥ ችግሮች ዙሪያ ለሚደረግ ጥናት የተዘጋጀ መጠይቅ፡- አማርኛ ቅጅ

| ተ.ቁ | ጥያቄዎች | | አማራጭ መልሶች | | ኮድ |  |
| --- | --- | --- | --- | --- | --- | --- |
| 1. ማህበራዊ እና ኢኮኖሚያዊ ጉዳዮችን በተመለከተ የተዘጋጁ ጥያቄዎች | | | | | |  |
| 101 | | እድሜ | ………………… ዓመት | |  |  |
| 102 | | የጋብቻ ሁኔታ | ያገባች  የፈታች  ባሏ የሞተባት  ያላገባች | | 1  2  3  4 |  |
| 103 | | የመጀመሪያ ጋብቻዎት እድሜ | __________ዓመት | |  |  |
| 104 | | ሀይማኖትዎ ምንድን ነዉ? | ኦርቶዶክስ ክርስቲያን  ሙስሊም  ፕሮቴስታንት  ካቶሊክ  ሌላ ( ይጠቀስ) --------------- | | 1  2  3  4  5 |  |
| 105 | | ብሔርዎ ምንድን ነዉ? | አማራ  ኦሮሞ  ትግሬ  ሌላ (ይጠቀስ) ------……. | | 1  2  3  4 |  |
| 106 | | የትምህርት ደረጃዎ? | ማንበብና መፃፍ የማትችል  ማንበብና መፃፍ የምትችል  መጀመሪያ ደረጃ ያጠናቀቁና በላይ | | 1  2  3 |  |
| 107 | | የቤተሰብ አስተዳዳሪ የተሰማራበት የስራ መስክ? | አርሶ አደር  ነጋዴ  ሌላ ካለ ይጠቀስ,___________ | | 1  2  3 |  |
| 108 | | የቤቱ አስተዳዳሪ ማን ነዉ | አባት  እናት  ሌላ ካለ ይጠቀስ | | 1  2  3 |  |
| 109 | | የቤት አስተዳዳሪ የትምህርት ደረጃ? | ማንበብና መፃፍ የማትችል  ማንበብና መፃፍ የምትችል  መጀመሪያ ደረጃ ያጠናቀቁና በላይ | | 1  2  3 |  |
| 110 | | ዓመታዊ የቤተሰብ ገቢ | ----------- ኩንታል | |  |  |
| 1. **አትክልትና ፍራፍራ ፍሬ** | | | | | |  |
| 201 | | አትክልትና ፍራፍሬ ትተክላላችሁ? | 1. አወ  2. የለም. ወደ ጥያቄ ቁጥር 3.1 ይለፉ | |  |  |
| 202 | | ምን ምን አትክልት ያዘምራሉ? | ቅጠላ ቅጠል  ፍራፍሬ  ሁለቱንም  ሌላ ካለ ይጠቀስ_____ | | 1  2  3  4 |  |
| 203 | | አትክልትና ፍራፍሬን ለምን አላማ ነዉ የሚተክሉት? | ለሽያጭ  ለቤት ፍጆታ  ለሁለቱም  ሌላ ካለ ይጠቀስ_________ | | 1  2  3  4 |  |
| 1. **የአመጋገብ ልምድን በተመለከተ ፡-**ባለፈዉ 24 ስዓት ዉስጥ ከዚህ በታች ከተዘረዘሩት የምግብ አይነቶች የወሰዱትና ያልወሰዱትን ለመለየት በየምግብ ምድቡ አንዱን ከወሰዱ አዎ ይበሉ፡ምንም ካልወሰዱ የለም ይበሉ | | | | | | |
|  | | የምግብ ምድብ | የምግብዝርዝር | | ኮድ |  |
| 301 | | እህል እና የህል ዉጤቶች | በቆሎ፣ስንዴ፤ዳጉሳ ወይም ሌላ ምግብ ከነዚህ የተዘጋጀ ለምሳሌ ዳቦ ወይም እንጀራ | | 1  2 |  |
| 302 | | በቫይታሚን ኤ የበለፀጉ አትክልቶችና ስሮች | ካሮት፣ስኳር ድንች ብርቱካን፣ፓፓያ | | 1  2 |  |
| 303 | | ግንዶችናስሮች | ድንች፤ቀይስር፤እና ሌሎች ምግቦች ከስር የተዘጋጁ | | 1  2 |  |
| 304 | | ደማቅ አረንጓዴ ተክሎች | ጎመን፣ጥቅል ጎመን. | | 1  2 |  |
| 305 | | ሌሎች አትክልቶች | ቲማቲም፤ሽንኩርት | | 1  2 |  |
| 306 | | ስጋ እና አሳ | ስጋ፤አሳ | | 1  2 |  |
| 307 | | እንቁላል | ዶሮ፤እንቁላል | | 1  2 |  |
| 308 | | ጥራጥሬ | ባቄላ,አተር፤ጓያ፤ሽምብራ | | 1  2 |  |
| 309 | | ወተትና የወተት ዉጤቶች | ወተት አይብ፤እርጎ | | 1  2 |  |
| 310 | | በቀን ስንት ጊዜ ይመገባሉ | ሶስት ጊዜ በታች  ሶስት ጊዜ  አራት ጌዜ  ከአራት ጊዜ በላይ | | 1  2  3  4 |  |
| 1. **የመጠጥ ዉሃ አቅርቦትንና የመፀዳጃ ፋሲሊቲን በተመለከተ** | | | | | |  |
| 401 | | የመጠጥ ዉሃ የሚያገኙት የት ነዉ? | - ከህዝብ የጋራ የቧንቧ ዉሃ - ከጎለበተ ምንጭ - ከወራጅ ዉሃ - ሌላ ካለ ይጠቀስ _______ | 1  2  3  4 | |  |
| 402 | | ዉሃ አክመዉ ይጠቀማሉ | አወ  የለም, | 1  2 | | 2 ከሆነ ወደጥያቄቁጥር. 404 |
| 403 | | አንዴት ነዉ ዉሃን የሚያክሙት? | በማፍላት  በማጥለል  ሌላ ካለ ይጠቀስ ______ | 1  2  3 | |  |
| 404 | | ቤትዎት ዉሃ ከሚገኝበት ቦታ በምን ያህል ደቂቃ ደርሶ መልስ ይወስዳል? | _________ ደቂቃ |  | |  |
| 405 | | በቀን ስንት ጊዜ ዉሃ ይቀዳሉ | አንድ ጊዜ  ሁለት ጊዜ  ሶስት ጊዜና በላይ | 1  2  3 | |  |
| 406 | | መጸዳጃ ቤት አለዎት? | አዎ  የለም | 1  2 | |  |
| 407 | | ከመፀዳጃ ቤት መልስ እጅዎትን ይታጠባሉ? | አዎ  የለም | 1  2 | |  |
| 408 | | መጸዳጃ ቤት ከሌለዎት የት ይጸዳዳሉ? | የጋራ መጸዳጃ ቤት  ሜዳ ላይ  ሌላ ካለ ይጠቀስ_________ | 1  2  3 | |  |
| 1. **ለእናት የሚደረግ ክትትልና ጤና ጋር ተያያዥ ጉዳዮችን በተመለከተ** | | | | | |  |
| 501 | | ስንት ጊዜ ነፍሰጡር ሆነዉ ያዉቃሉ? | ----------- | |  |  |
| 502 | | ስንት ልጅ አለዎት? | ------------- | |  |  |
| 503 | | ልጅ ካለዎት የመጨረሻ ልጅዎት እደሜ? | ------------ዓመት | |  |  |
| 504 | | በነፍሰ-ጡርነትዎ ወቅት ክትትል ነበረዎት | 1. አወ 2. የለም | |  |  |
| 505 | | የቤተሰብ ብዛት? | ___________ | |  |  |
| 506 | | ዘመናዊ የወሊድ መቆጣጠሪያ ይጠቀማሉ | 1. አወ 2. የለም | |  |  |
| 507 | | ባለፈዉ አንድ ወር ዉስጥ አሞዎት ያዉቃል? | አወ  አያዉቅም , | | 1  2 | የለምከሆነወደጥያቄቁጥርበ 601ይለፉ. |
| 508 | | ለምን ያህል ጊዜ አሞዎት ነበር? | ከ 1 ሳምንት በታች  1-2 ሳምንት  3-4 ሳምንት  ከዛ በላይ | | 1  2  3  4 |  |
| 509 | | ከህመምዎት መቼ አገገሙ? | በዚህሳምንት ዉሥጥ  ከሁለት ሳምንት በፊት  ከሶስት ሳምንት በፊት  ሌላ ካለ ይገለጽ_______ | | 1  2  3  4 |  |
| 1. **Anthropometric data** | | | | | |  |
| 601 | | ክብደት በኪሎ ግራም | ልኬታ1_____ኪ.ግ፣  ልኬታ2________ኪ.ግ | |  |  |
| 602 | | ቁመት በሜትር | ልኬታ1_____ሜ፣  ልኬታ2____ሜ | |  |  |
