# Supplementary material for: Determinants of chronic energy deficiency among non-pregnant and non-lactating women of reproductive age in rural Kebeles of Dera District, North West Ethiopia, 2019: Unmatched case control study
Source: PLoS One. 2020 Oct 29;15(10):e0241341. doi: 10.1371/journal.pone.0241341 (PMC7595398; doi:10.1371/journal.pone.0241341)
Supplement: Attachment — Submitted filename: Response to Reviewers.docx [file pone.0241341.s002.docx]

**Response to Reviewers**

Academic editor:

1. Please discuss whether and how the questionnaire was validated. If this did not occur, please provide the rationale for not doing so.

- The questionnaire was adapted after reviewing previous literatures done on similar title. We have cited the references in line -109. Anthropometric measurements (height and weight) were done based on WHO standards (line 121). DDS of the study participants was collected based on Food and agriculture organization (FAO) guide line to measurement minimum dietary diversity for women(line 111- 115).

2. Discuss the following publication: <https://bmcnutr.biomedcentral.com/articles/10.1186/s40795-015-0005-y>.

The above publication is used and discussed in the manuscript. Check line 226. In this study, meal frequency was one determinant factor for CED among study participants.

3. Regarding participant consent (<18 years), Participants were informed that they have the full right to refuse to participate in the study or can interrupt/withdraw if they want. Confidentiality of the information was assured and the privacy of the study participants was respected and kept as well. Written informed consent was obtained from each study participant and/or from parents/guardians of <18 years old study participants (line 148-152).

Reviewer #1:

1. Line 120-121: what is the basis for considering women with a BMI of less than 18.5 kg/m2 as chronic energy deficiency? Are there any references?

- According to the report of a working party of the International Dietary Energy Consultative Group, BMI of less than 18.5 kg/m2 among adults is considered as chronic energy deficiency. I have cited the reference in line 122-123.

2. Tables 4, Does presence of home gardening affect dietary habits? Did the author collect other dietary habits such as hand washing before eating, with regular quantitative meal daily or not?

- We have check multicollinearity between all independent variables. Variance inflation factor was (VIF) below 10. Therefore, presences of home gardening do not affect dietary habits (line 140-142).
- We did not collect information on hand washing before eating and portion size of meal. We have mentioned these as a limitation (line 258-260). We rather collect information on access to safe drinking water and latrine facility including hand washing after toilet. We indirectly try to assess the hand washing habit of the study participants (S3- questionnaire).
